# Global attitudes in the management of acute appendicitis during COVID‐19 pandemic: ACIE Appy Study

**DOI:** 10.1002/bjs.11999

**Published:** 2020-10-08

**Authors:** B Ielpo, M Podda, G Pellino, F Pata, R Caruso, G Gravante, S Di Saverio, ACIE Appy Study Collaborative

**Affiliations:** 1 Department of Surgery, Hepatopancreatobiliary Unit, University Hospital Leon, Leon, Spain; 2 Colorectal Surgery, Vall d'Hebron University Hospital, Barcelona, Spain; 3 Hospital Universitario HM Sanchinarro, Madrid, Spain; 4 Department of General and Emergency Surgery, Cagliari University Hospital, Azienda Ospedaliero‐Universitaria, Cagliari, Italy; 5 Department of Advanced Medical and Surgical Sciences, Universitá degli Studi della Campania ‘Luigi Vanvitelli’, Naples, Italy; 6 General Surgery Unit, Nicola Giannettasio Hospital, Corigliano‐Rossano, Italy; 7 Department of General Surgery, La Sapienza University, Rome, Italy; 8 Department of General Surgery, Ospedale ‘Francesco Ferrari’, Casarano, Italy; 9 Department of General Surgery, University of Insubria, University Hospital of Varese, ASST Sette Laghi, Regione LombardiaVarese, Italy

## Abstract

**Background:**

Surgical strategies are being adapted to face the COVID‐19 pandemic. Recommendations on the management of acute appendicitis have been based on expert opinion, but very little evidence is available. This study addressed that dearth with a snapshot of worldwide approaches to appendicitis.

**Methods:**

The Association of Italian Surgeons in Europe designed an online survey to assess the current attitude of surgeons globally regarding the management of patients with acute appendicitis during the pandemic. Questions were divided into baseline information, hospital organization and screening, personal protective equipment, management and surgical approach, and patient presentation before *versus* during the pandemic.

**Results:**

Of 744 answers, 709 (from 66 countries) were complete and were included in the analysis. Most hospitals were treating both patients with and those without COVID. There was variation in screening indications and modality used, with chest X‐ray plus molecular testing (PCR) being the commonest (19·8 per cent). Conservative management of complicated and uncomplicated appendicitis was used by 6·6 and 2·4 per cent respectively before, but 23·7 and 5·3 per cent, during the pandemic (both *P* < 0·001). One‐third changed their approach from laparoscopic to open surgery owing to the popular (but evidence‐lacking) advice from expert groups during the initial phase of the pandemic. No agreement on how to filter surgical smoke plume during laparoscopy was identified. There was an overall reduction in the number of patients admitted with appendicitis and one‐third felt that patients who did present had more severe appendicitis than they usually observe.

**Conclusion:**

Conservative management of mild appendicitis has been possible during the pandemic. The fact that some surgeons switched to open appendicectomy may reflect the poor guidelines that emanated in the early phase of SARS‐CoV‐2.

## Teaser

The COVID‐19 pandemic required reorganization of surgical services, affecting patients with common surgical diseases including acute appendicitis. No evidence is available on the topic. This study found global variation in screening policies, use of personal protective equipment and intraoperative directives. There has been increased adoption of non‐operative management and open appendicectomy.

Hands off

## Resumen

**Antecedentes:**

Las estrategias quirúrgicas están siendo adaptadas en presencia de la pandemia de la COVID‐19. Las recomendaciones del tratamiento de la apendicitis aguda se han basado en la opinión de expertos, pero hay muy poca evidencia disponible. Este estudio abordó este aspecto a través de una visión de los enfoques mundiales de la cirugía de la apendicitis.

**Métodos:**

La Asociación de Cirujanos Italianos en Europa (ACIE) diseñó una encuesta electrónica en línea para evaluar la actitud actual de los cirujanos a nivel mundial con respecto al manejo de pacientes con apendicitis aguda durante la pandemia. Las preguntas se dividieron en información basal, organización del hospital y cribaje, equipo de protección personal, manejo y abordaje quirúrgico, así como las características de presentación del paciente antes y durante de la pandemia. Se utilizó una prueba de ji al cuadrado para las comparaciones.

**Resultados:**

De 744 respuestas, se habían completado 709 (66 países) cuestionarios, los datos de los cuales se incluyeron en el estudio. La mayoría de los hospitales estaban tratando a pacientes con y sin COVID. Hubo variabilidad en las indicaciones de cribaje de la COVID‐19 y en la modalidad utilizada, siendo la tomografía computarizada (CT) torácica y el análisis molecular (PCR) (18,1%) las pruebas utilizadas con más frecuencia. El tratamiento conservador de la apendicitis complicada y no complicada se utilizó en un 6,6% y un 2,4% antes de la pandemia frente a un 23,7% y un 5,3% durante la pandemia (*P* < 0.0001). Un tercio de los encuestados cambió la cirugía laparoscópica a cirugía abierta debido a las recomendaciones de los grupos de expertos (pero carente de evidencia científica) durante la fase inicial de la pandemia. No hubo acuerdo en cómo filtrar el humo generado por la laparoscopia. Hubo una reducción general del número de pacientes ingresados con apendicitis y un tercio consideró que los pacientes atendidos presentaban una apendicitis más grave que las comúnmente observadas.

**Conclusión:**

La pandemia ha demostrado que ha sido posible el tratamiento conservador de la apendicitis leve. El hecho de que algunos cirujanos cambiaran a una apendicectomía abierta podría ser el reflejo de las pautas deficientes que se propusieron en la fase inicial del SARS‐CoV2.

## Introduction

Since the first cases of an unusual pneumonia were described in China during late December 2019, the new coronavirus, severe acute respiratory syndrome coronavirus 2 (SARS‐CoV‐2), which causes COVID‐19, has spread rapidly worldwide. On 11 March 2020, COVID‐19 disease was declared a pandemic infection by the WHO. As of 2 June 2020, 6 194 533 confirmed cases and 376 320 deaths have been reported globally^1^.

Healthcare systems adopted specific measures to preserve hospital capacity, increase ICU beds and create COVID‐19 units, including the postponement of all non‐oncological elective procedures^2^. Furthermore, in light of preliminary data^3^ reporting a high perioperative mortality rate (20·5 per cent) among patients operated in the incubation phase of COVID‐19, several surgical societies^4–6^ globally recommended a safe approach even in emergency surgery, with implementation of non‐operative management (NOM) whenever possible, including for acute appendicitis. Other recommendations included selective use of minimally invasive surgery (MIS), and the use of ultrafiltration systems for carbon dioxide filtering and evacuation during laparoscopy^2^^,^^3^^,^^6^^,^^7^. However, given the lack of availability of ultrafiltration systems, the paucity of personal protective equipment (PPE), the shortage of surgical workforce, and the impossibility of routine testing of all patients, a trend towards a more conservative attitude may have occurred during the COVID‐19 pandemic.

Approximately 300 000 people undergo appendicectomy annually in the USA^8^. According to a recent meta‐analysis^9^ on the topic, the most recently reported incidence of acute appendicitis is approximately 98 per 100 000 individuals per year in the USA. Therefore, it could be estimated that around 322 000 patients might have suffered from acute appendicitis in 2019 in the USA^10^. In other words, should the state of emergency last 2 months, in the USA alone, approximately 54 000 patients would be affected, which would rise to 80 000 in the event of prolongation of the state of emergency for an additional month.

Given the rapidly evolving situation and the absence of evidence to support recommendations during the COVID‐19 pandemic, it is useful to assess how the current situation is influencing the management of patients with acute appendicitis, as no definitive conclusions can be drawn at present.

The aim of this global, multicentre study survey was to explore whether the strategies for management and choice of surgical approach for patients admitted for acute appendicitis have changed during the pandemic among a large pool of respondents from several countries, and, if so, how.

## Methods

The Association of Italian Surgeons in Europe (Associazione Chirurghi Italiani in Europa, ACIE) working group conducted an internet‐based survey to investigate how the COVID‐19 pandemic changed the clinical decision for patients with acute appendicitis. The data sample came from different surgeons and trainees working in general surgery units across Europe, Asia, Africa, Oceania, North and South America. Survey respondents were informed of the purpose of the study and their participation remained voluntary as no incentives were offered to participants.

### Questionnaire development and composition

The Steering Committee developed the questionnaire using web‐based and remote discussion and brainstorming, after identifying the components and topics to include. The technical functionality of the electronic questionnaire was tested before the invitations were sent. Baseline information on respondents, along with names and locations of surgical units, were stored with the questionnaire. Once agreement had been reached, the questionnaire (The COVID‐19 Appy Study Form) was completed using Google Form survey software (Google, Mountain View, California, USA).

The questionnaire has five sections and includes 40 questions (*Table S1*, supporting information). Only closed‐ended questions were used. The first four sections include general questions about the hospital organization, screening policies, PPE, and personal attitudes about the management of acute appendicitis. The final section focuses on the real‐life analysis of presentation and management strategies for patients with acute appendicitis before and during the COVID‐19 pandemic.

Uncomplicated appendicitis was defined as appendicitis without an abscess, whereas complicated appendicitis included the presence of an intra‐abdominal abscess. NOM was defined as conservative management with antibiotics; this could include percutaneous abscess drainage.

The list of alternatives for each quantitative question included the following categories: 25 per cent or less, 26–50, 51–75 and 76–100 per cent. The Steering Committee decided to use ranges of predetermined percentages to allow easier aggregation and analysis of the information collected.

The estimated time needed to complete the survey was 8–10 min. The aim was to define the current status of the management of acute appendicitis compared with that in the interval before the pandemic. The respondents were invited to disclose their hospital and country of practice.

**Fig. 1:**
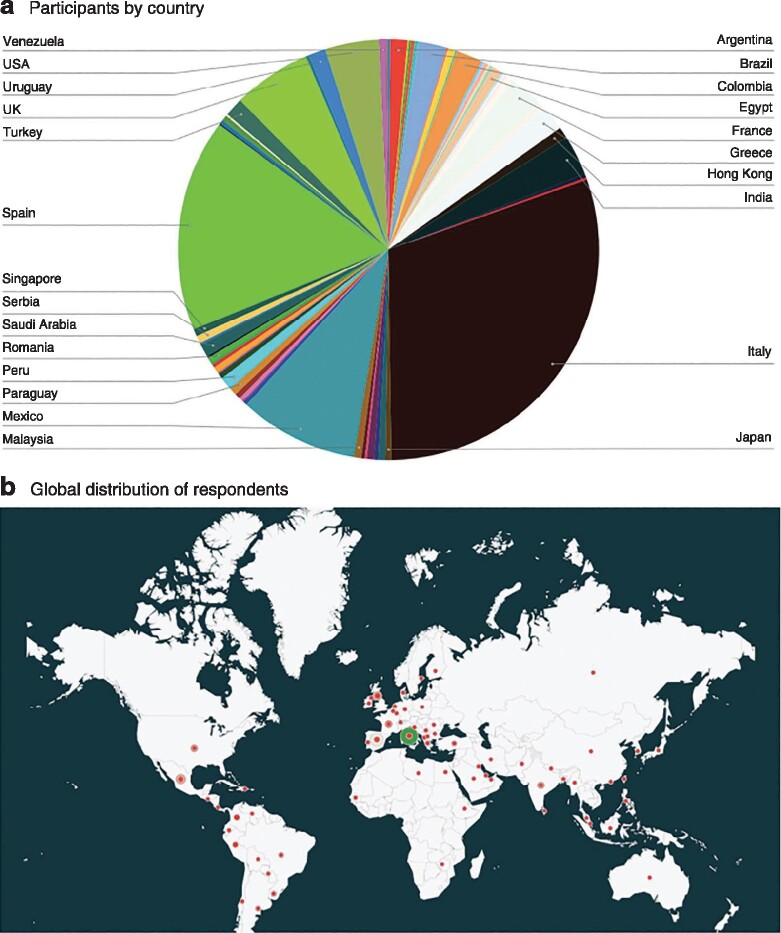
Breakdown of countries of origin of participants in the study

**a** Chart showing percentage of participants from each country and **b** map showing global distribution of respondents.

### Study circulation

On 8 April 2020, the questionnaire was made available online and was open for completion until 15 April 2020. The link (https://docs.google.com/forms/d/e/1FAIpQLSfVelIe3yrEZRZx5FebUYMCrxzC3WqYi3GNnOuN8jjRPyO9ZA/formResponse) was circulated by means of personal e‐mail invitations, and was shared on social media (LinkedIn, Twitter, Facebook, WhatsApp groups) by members of the Steering Committee.

### Data handling and extraction

A member of the Steering Committee downloaded the questionnaires and shared them with the other members for data analysis and discussion. Multiple entries from the same individual or members of the same surgical unit were sought manually and eliminated if contradictory findings were observed.

### Statistical analysis

Categorical variables are reported using counts and percentages. Data from the surveys were compared using 4 × 2 contingency tables and analysed by means of the χ^2^ test. *P* < 0·050 was considered statistically significant. SPSS® version 22 (IBM, Armonk, New York, USA) was used for the statistical analysis.

## Results

**Fig. 2:**
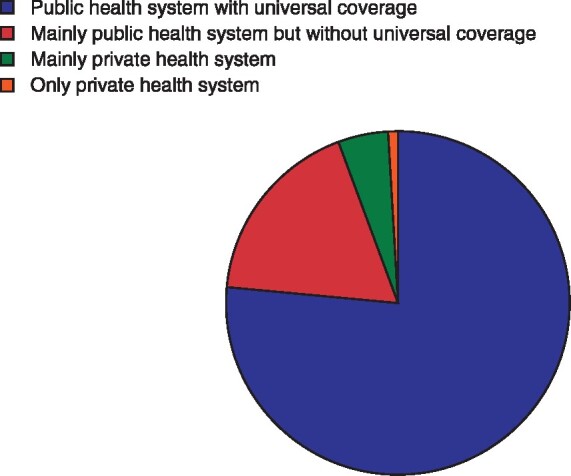
Representation of national health systems of participants.

Overall, 744 answers were received; after removing those that were incomplete, 709 were included from 66 countries. The distribution of respondents by country of origin is shown in *Fig*. *1*. Most respondents were from countries that were the most affected at the time of the survey; almost half of the answers were returned from Spain and Italy. Some 69·9 per cent of respondents were consultant/attending surgeons, 22·7 per cent trainees/residents and 6·9 per cent fellows. General surgeons had a higher rate of participation (57·6 per cent) than colorectal (22·9 per cent), hepatopancreatobiliary (9·9 per cent), upper gastrointestinal (6·3 per cent) and paediatric (3·3 per cent) surgeons (*Table S2*, supporting information). Baseline information about the national health system and type of hospital in which each of the survey participants reported working is shown in *Fig*. *2*.

### Hospital organization and screening policies

Some 8·9 per cent of participants declared that their hospital was exclusively dedicated to patients with COVID‐19, whereas 83·1 per cent reported restricted COVID‐19 areas, and 8·0 per cent do not treat patients with COVID‐19. The majority of respondents (51·0 per cent) reported that only patients with respiratory symptoms or suspected of having SARS‐CoV‐2 infection are screened before surgery for acute appendicitis; 37·4 per cent routinely screen all patients before surgery, whereas 11·6 per cent of respondents declared that they do not test under any circumstances.

Surgeons who stated that they screen patients with acute appendicitis before surgery adopted the following protocols: chest X‐ray (7·3 per cent), chest X‐ray and serology (6·3 per cent), chest X‐ray and PCR (19·8 per cent), chest CT (13·9 per cent), chest CT and serology (6·7 per cent), chest CT and PCR (18·1 per cent), serology alone (1·4 per cent), PCR alone (17·2 per cent) and rapid test (9·3 per cent).

Overall, 28·2 per cent of respondents reported that patients tested positive for SARS‐CoV‐2 after surgery, with 21·3 per cent reporting that this occurred in 1–5 per cent of patients at their centre, 2·8 per cent in 6–10 per cent, and 4·1 per cent in more than 10 per cent of patients.

Screening policies according to the country in which the respondents practise are shown in *Appendix S2* (supporting information). In Spain, the UK and Italy, more than 50 per cent of respondents screened all patients, irrespective of clinical symptoms. In other countries, such as Brazil, the USA, Mexico and France, the most frequent trend has been to test patients only in the presence of respiratory symptoms; 17·2 per cent of respondents from the USA, 35·9 per cent from Mexico, 15·8 per cent from France and 13·3 per cent from Brazil did not routinely screen patients with appendicitis for SARS‐CoV‐2.

### Personal protective equipment

*Table* *1* shows changes in the use of PPE. Most surgeons (37·9 per cent) did not change their use of PPE in COVID‐19‐negative patients; the remainder adopted some measures that are not usually used, the commonest being use of face masks and goggles (24·0 per cent). In COVID‐19‐positive patients, 4·1 per cent of surgeons stated that no changes were adopted for operative protection, 4·3 per cent reported use of an FFP2/FFP3 face mask, 1·9 per cent an N95 face mask, 0·4 per cent goggles, 56·3 per cent a FFP2/FFP3 face mask and goggles, and 33·0 per cent an N95 face mask and goggles.

**Table 1.** Changes in use of personal protective equipment during COVID‐19 pandemic, according to patient SARS‐CoV‐2 status

	% of respondents		
	Patients who tested negative for COVID‐19	Patients not tested for COVID‐19	Patients who tested positive for COVID‐19
No changes	37·9	18·1	4·1
FFP2/FFP3 face mask	10·2	10·6	4·3
N95 face mask	6·4	6·0	1·9
Goggles	3·4	2·4	0·4
FFP2/FFP3 face mask and goggles	24·0	40·1	56·3
N95 mask and goggles	18·0	22·6	33·0

**Fig. 3:**
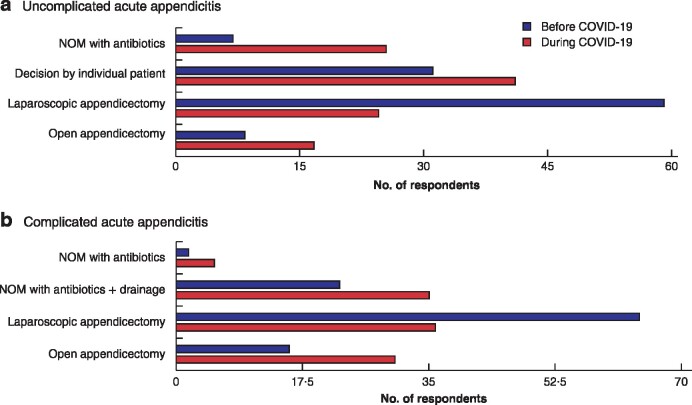
Management of uncomplicated and complicated acute appendicitis before and during COVID‐19 pandemic

**a** Uncomplicated and **b** complicated acute appendicitis. NOM, non‐operative management.

In treatment of COVID‐19 patients who were not tested for COVID‐19, 40·1 per cent reported using an FFP2/FFP3 face mask and goggles, 22·6 per cent an N95 face mask and goggles, 10·6 per cent an FFP2/FFP3 mask, 6 per cent an N95 mask and 2·4 per cent goggles alone; 18·1 per cent did not use PPE.

### Personal attitude: operative *versus* non‐operative management of acute appendicitis

In patients with uncomplicated appendicitis (no right iliac fossa abscess), 28·5 per cent of the surgeons changed their attitude during the COVID‐19 pandemic: of these, 15·6 per cent did so in COVID‐19‐positive and untested patients, and only 13·2 per cent in COVID‐19‐positive patients; 42·7 per cent did not change their conduct at all. In the event of appendicitis complicated by right iliac fossa abscess, 24·6 per cent changed their attitude only in COVID‐19‐positive patients and 47·1 per cent did not change their attitude at all. Approximately 22 per cent of the respondents declared that they would change their attitude from surgery to NOM with antibiotics, or vice versa, if they had the chance to test all patients before surgery; 17·5 per cent stated that they already test all patients, whereas 26·9 per cent stated that they would have changed their attitude only if quick tests or PCR were available.

Before the COVID‐19 pandemic, 6·6 per cent of the respondents adopted NOM with antibiotics for patients with uncomplicated acute appendicitis, compared with 23·7 per cent during the pandemic (*P* < 0·001) (*Table* *2*). Regarding complicated acute appendicitis, NOM was used by 2·4 and 5·3 per cent before and during the pandemic, and percutaneous drainage by 21·1 *versus* 32·9 per cent, respectively (*P* < 0·001) (*Table* *2* and *Fig*. *3*).

**Table 2:** Patient presentation and management of acute appendicitis before and during COVID‐19 pandemic

	% of respondents		
	Before COVID‐19	During COVID‐19	*P* *
**How do you manage uncomplicated acute appendicitis (no abscess)?**			< 0·001
Non‐operative management with antibiotics	6·6	23·7	
Decision by individual patient	29·0	38·8	
Straightforward laparoscopic appendicectomy	57·2	22·5	
Straightforward open appendicectomy	7·2	15·0	
**How do you manage complicated acute appendicitis (abscess)?**			< 0·001
Non‐operative management with antibiotics	2·4	5·3	
Non‐operative management with antibiotics and percutaneous drainage	21·1	32·9	
Straightforward laparoscopic appendicectomy	62·5	33·7	
Straightforward open appendicectomy	14·0	28·1	
**How many patients with acute appendicitis are referred to your hospital per month?**			< 0·001
< 5	13·3	39·3	
5–9	26·9	33·5	
10–20	27·0	16·7	
> 20	32·8	10·5	
**In what percentage of patients with uncomplicated acute appendicitis (no abscess) is non‐operative management with antibiotics used at your hospital?**			< 0·001
≤ 25	79·3	60·1	
26–50	11·8	16·2	
51–75	6·6	11·6	
76–100	2·3	12·1	
**What percentage of patients with uncomplicated acute appendicitis (no abscess) treated conservatively with antibiotics are sent home and followed up at the outpatient clinic at your hospital?**			< 0·001
≤ 25	78·2	67·5	
26–50	10·9	12·9	
51–75	5·7	9·8	
76–100	5·2	9·8	
**What percentage of patients with complicated acute appendicitis (abscess) undergo conservative treatment with antibiotics +/– percutaneous drainage at your hospital?**			0·001
≤ 25	77·3	68·4	
26–50	10·8	12·5	
51–75	5·6	9·3	
76–100	6·3	9·8	
**What percentage of patients with acute appendicitis treated with surgery undergo open appendicectomy at your hospital?**			< 0·001
≤ 25	73·6	53·8	
26–50	8·7	13·7	
51–75	8·6	9·8	
76–100	9·1	22·7	

* χ^2^ test.

### Personal attitude: surgical approach

**Fig. 4:**
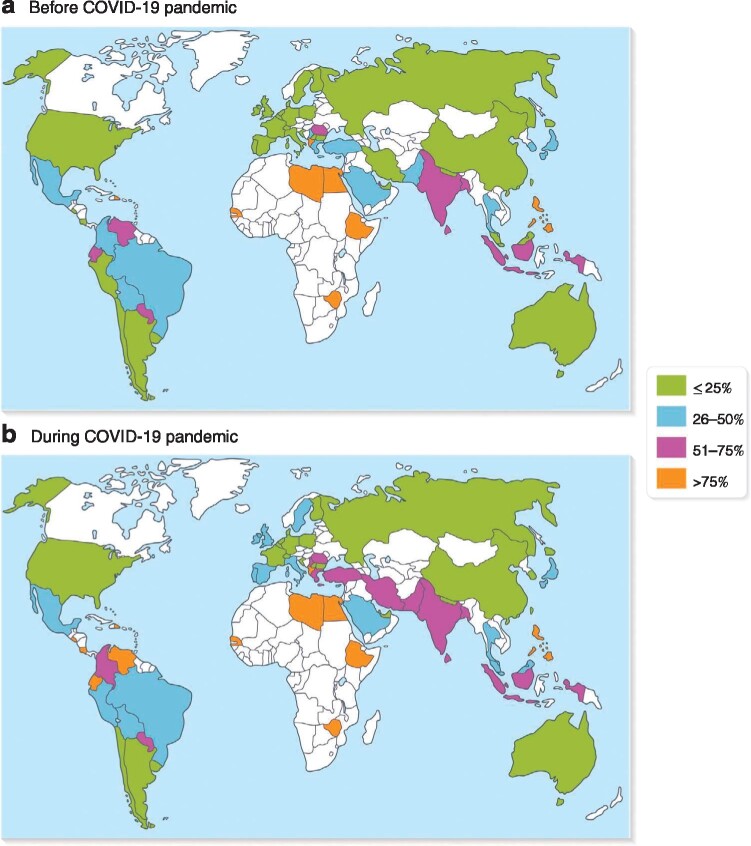
Use of open appendicectomy before and during COVID‐19 pandemic

A total of 39·0 per cent of respondents changed their standard surgical approach from laparoscopic to open (36·6 per cent) or from open to laparoscopic (2·4 per cent) during the pandemic. *Fig*. *4* shows how the rate of open appendicectomy changed from before to during the pandemic globally.

Values are mean number of participants performing open appendicectomy.

The preferred surgical approach and associated safety measures being adopted are summarized in *Table* *3*. Some 30·1 and 28·0 per cent of surgeons prefer open appendicectomy in COVID‐19‐positive and untested patients respectively. Specific devices to filter surgical plumes are used by 43·0 per cent of respondents in COVID‐19‐positive and by 17·0 per cent in untested patients, whereas no filtering systems for carbon dioxide are being used in 6·2 per cent and 49·4 per cent respectively. If any smoke evacuation system with filters is being used, 32·8 per cent of surgeons use commercially available systems (*Table* *3*).

**Table 3.** Surgical approach for acute appendicitis and aspiration of smoke plumes

	% of respondents	
	COVID‐19 positive	Untested patients
**Surgical approach**		
Always open surgery, personal preference	30·1	28·0
Laparoscopic surgery without specific devices for protection and smoke evacuation	6·2	49·4
Laparoscopic surgery with specific devices for protection and smoke evacuation	43·0	17·0
I would use laparoscopy, but do not have devices for pneumoperitoneum/smoke evacuation	20·7	5·6
**Systems to filter surgical smoke**		
If laparoscopic appendicectomy is performed, do you use any filter system?		
Yes	37·8	
Yes, only in COVID‐19‐positive patients	11·9	
Yes, only in COVID‐19‐positive or untested patients	24·3	
No	26·0	
If any smoke evacuation system is used, which type of device do you use?		
Commercially available	32·8	
Commercially available with filtration connected to a container with water	7·7	
Commercially available with filtration connected to a sealed container	22·0	
Home‐made	11·9	
Home‐made with filtration connected to a container with water	14·0	
Home‐made with filtration connected to a sealed container	11·6	

A straightforward open appendicectomy for uncomplicated appendicitis was used by 7·2 per cent of participants before and 15·0 per cent during the pandemic; for complicated appendicitis, this approach was used by 14·0 per cent before and 28·1 per cent during the pandemic (*P* < 0·001) (*Table* *2* and *Fig*. *3*).

In all, 76·4 per cent of surgeons who took part in the survey were confident in performing open appendicectomy, whereas 15·8 per cent preferred supervision by someone with experience in open appendicectomy.

### Patient presentation before and during pandemic at participants' institutions

**Fig. 5:**
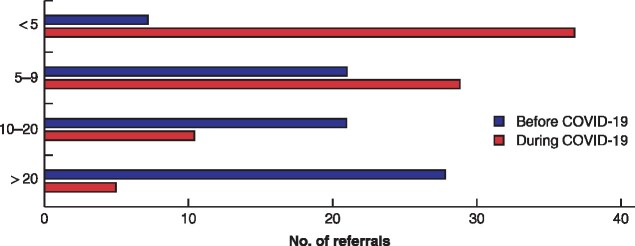
Hospital referrals for acute appendicitis before and during the COVID‐19 pandemic at participants' institutions.

Before the pandemic, 32·8 per cent of surgeons stated that more than 20 patients per month were usually referred to their hospital with acute appendicitis, compared with only 10·5 per cent reported during the pandemic (*P* < 0·001) (*Table* *2* and *Fig*. *5*). According to 34·3 per cent of participants, patients had more advanced disease features at presentation during the COVID‐19 emergency.

Only 8·9 per cent of the respondents reported that NOM was being used in over half of procedures for uncomplicated appendicitis at their institution before compared with 23·7 per cent during the COVID‐19 pandemic (*P* < 0·001) (*Table* *2*). The percentage of respondents reporting that their institution treated uncomplicated appendicitis with antibiotics at home and followed up at the outpatient clinic in almost all patients (76–100 per cent) increased from 5·2 per cent before to 9·8 per cent during the pandemic (*P* < 0·001). Similar trends in use of NOM with antibiotics with or without percutaneous drainage were observed in patients with complicated appendicitis, with 11·9 *versus* 19·1 per cent of the respondents' institutions using it in more than 50 per cent of cases before *versus* during the pandemic (*P* = 0·001) (*Table* *2*).

Regarding surgical technique, the proportion of centres using open appendicectomy in more than 50 per cent of patients increased from 17·7 to 32·5 per cent during the pandemic (*P* < 0·0001) (*Table* *2*).

### Subgroup analyses

Country‐specific subgroup analyses are shown in *Appendix S2* (supporting information).

## Discussion

Given the lack of available data about the management of acute appendicitis during the COVID‐19 pandemic, the authors decided to conduct the first worldwide survey about its current management. This survey showed a high degree of variation among the policies used for screening patients (indications and modalities) with acute appendicitis, as well as different attitudes to management of the condition.

Since the outbreak of COVID‐19 pandemic in Europe, several guidelines and recommendations^4^^,^^6^^,^^11–13^ have been released to support the decision‐making process in surgery. The overall level of evidence is low, with many recommendations based on expert opinion and case series. Even though substantial agreement exists on many issues, some aspects remain controversial.

Delivering a surgical service in a safe manner is a key factor in the response to a pandemic. According to this survey, 18·1 per cent of surgeons have not changed their use of PPE when treating untested patients, and 4·1 per cent are not using protective measures even for COVID‐19‐positive patients. These figures might be justified by the shortage of PPE. Of note, 37·9 per cent of surgeons have not changed their PPE when treating COVID‐19‐negative patients, which is reasonable given the possibility of false‐positive results. The results confirm the current uncertainty concerning PPE use in the context of the COVID‐19 crisis.

The availability of PPE can influence the perceived safety and fears of surgeons working under such stressful conditions^14^. This should be addressed in detail, considering that some countries have not yet reached the peak of the pandemic and additional waves of COVID‐19 have been anticipated in the near future.

Most hospitals have been treating both patients with COVID‐19 and those without, but the screening policies for patients with appendicitis vary widely between centres. Screening all emergency patients for SARS‐CoV‐2 is advisable before surgery, whenever possible^4^. However, half of the respondents are only screening patients with respiratory symptoms or suspected infection. This raises concerns, as data on asymptomatic patients suggest that postoperative outcomes are poor, with high complication and mortality rates^3^. Approximately 12 per cent of participants have not been screening emergency patients at all. This is deeply worrisome, considering that 28·2 per cent of respondents reported that at least one patient tested positive after surgery, and this occurred in more than 10 per cent of cases according to 4·1 per cent of respondents. Furthermore, given the recently reported data that patients with COVID‐19 may have a worse postoperative outcome, it is paramount to test patients before any surgery, especially in an emergency setting where the risk of complications may be increased^3^. The high proportion of respondents from countries such as Mexico and the UK that did not test patients routinely might have been responsible for the course of COVID‐19 observed in these countries (*Appendix S2*, supporting information).

Guidelines for screening and testing continue to evolve as knowledge of the pandemic improves and the availability of testing kits increases. According to the latest Chinese guidelines^15^, the diagnosis of COVID‐19 must be confirmed by one of the following: real‐time reverse transcriptase–PCR; viral gene identified by gene sequencing highly homologous with SARS‐CoV‐2; or SARS‐CoV‐2‐specific IgM and IgG. Several studies have suggested that the majority of patients develop an antibody response only in the second week after onset of symptoms, thereby limiting the usefulness of antibody testing for early diagnosis^16^. The role of chest CT is debated. The American College of Radiology^17^ recommends not using chest CT for screening COVID‐19, and reserving it for hospitalized patients, when needed for management. Some societies recommend against the use of chest CT in screening for COVID‐19^18^, whereas others suggest that it can be used in emergency settings when it is not possible to wait for the results of a PCR test^4^. When assessing screening modalities, disagreement was noted among respondents. Most participants used chest X‐ray plus PCR (19·8 per cent) or PCR alone (17·2 per cent). Some 13·9 per cent used only chest CT, whereas 7·3 per cent used chest X‐ray alone. Clearer guidance about testing is desirable.

Although there is no evidence that SARS‐CoV‐2 could spread by aerosolization by both pneumoperitoneum and smoke during MIS, the risk cannot be ruled out at present. Some data on hepatitis B virus (HBV)‐positive patients suggested that HBV could be detected in surgical smoke during MIS^19^. Even if the risk is hypothetical with SARS‐CoV‐2, some have suggested that this should be prioritized over the benefits of laparoscopy. These considerations justify some discrepancies among current guidelines. Contradictions can be found in recommendations from the same surgical society; the American College of Surgeons has emphasized the benefits of laparoscopic appendicectomy as an outpatient procedure in patients with failed NOM in a guideline^12^, but suggested that laparoscopy should be avoided in another document about the optimal protection for surgeons^20^.

On the other hand, the current British Intercollegiate General Surgery^11^ guidance on COVID‐19 suggests that laparoscopy should be considered only in selected patients in whom the clinical benefit for the patient substantially outweighs the risk of potential viral transmission. Whenever possible, NOM should be considered; open appendicectomy is recommended if NOM is not feasible. Because ultrafiltration devices can be difficult to implement, erring on the side of safety may be the best option in the current situation^21^.

The benefits of laparoscopic appendicectomy should also be considered, including the possibility of performing surgery as an outpatient procedure^22^, shorter hospital stay, lower incidence of surgical‐site infections and faster recovery compared with the open technique^23^^,^^24^. These are promising features during an outbreak, where hospital capacity and resources are limited. Interestingly, most of the respondents did not change their attitude to the management of acute appendicitis, but approximately one in three changed the approach from laparoscopic to open. Almost one‐third of the participants reported performing open appendicectomy in all patients with COVID‐19, but 43·0 per cent of these would use laparoscopy if the devices for smoke filtering were available at their centre. Special attention should be paid to the establishment and evacuation of pneumoperitoneum, and liberal use of suction devices to remove smoke and aerosol during operations by means of an ultrafiltration system (smoke evacuation or filtration), especially before converting from laparoscopy to open surgery^25^^,^^26^. Moreover, intraoperative pneumoperitoneum pressure and carbon dioxide ventilation should be kept at the lowest possible levels without compromising exposure of the surgical field in order to minimize the effect of pneumoperitoneum on lung function and circulation, in an effort to reduce susceptibility to pathogens. Incisions for ports should be as small as possible to avoid leakage around ports^6^. Among those using systems to filter smoke, less than one‐third of respondents reported using commercially available devices.

However, 26·0 per cent reported that they are performing laparoscopic appendicectomy with no devices to filter carbon dioxide (*Table* *3*). Half of the respondents (49·4 per cent) use laparoscopy in untested patients. The finding that 4·1 per cent of respondents declared they had not changed their operational protection measures in COVID‐19‐positive patients is a finding that deserves thorough reflection. The importance of applying adequate measures is highlighted by the fact that almost 30 per cent of patients tested positive after surgery in the present study. Filtering the pneumoperitoneum through filters able to remove most viral particles is highly recommended^25^. Considering that COVID‐19 virus particles range in size from 0·06 to 0·14 μm, surgeons might be aware that not all smoke filters can effectively filter them. Ultralow particulate air (ULPA) filters are extremely efficient at filtering SARS‐CoV‐2. According to the ISO standard 29463 (issued to harmonize European Standard EN 1822 and US MIL‐STD‐282), a ULPA filter must have at least 99·9995 per cent efficiency at filtering particles with a most penetrating particle size (MMPS) of 0·12 μm. The MMPS is the particle at which the filter is less efficient. Smaller particles are filtered with even lower efficiency. Therefore, the authors' advice is to check that the filter is appropriate (0·1 μm) before undertaking laparoscopic surgery as well as performing a test of insufflation and smoke evacuation before use. Appropriate equipment and understanding are paramount in mitigating the risk of aerosolization.

It is worth considering that evacuation of smoke might be easier with laparoscopy than with open surgery^4^, if adequate measures are adopted. Although the evidence is poor, there are some concerns that the risk of virus aerosolization is higher during the open approach as smoke generated from electrocautery is more difficult to capture. Very few respondents reported a change to their usual management from open to laparoscopic appendicectomy. Conversely, the proportion of centres that performed 76–100 per cent of appendicectomies by an open approach increased from 9·1 per cent before to 22·7 per cent during the pandemic.

A potential issue that has been raised recently is the ability of training programmes to provide the skills to perform open appendicectomy proficiently and safely during recent years^27^. Most of the respondents were confident in performing open appendicectomy, excluding the possibility that this factor might have influenced their decision.

Also to be taken into consideration is the finding that there might have been a reduction in the number of patients admitted to emergency departments during the pandemic; according to the present survey, 13·3 per cent of centres had fewer than five patients with appendicitis referred per month before *versus* 39·3 per cent during the pandemic. Moreover, there may have been a trend towards more advanced presentation (34·2 per cent stated that this was the case, whereas 39·3 per cent were unsure). These factors might also play a role in the decision‐making between open and MIS appendicectomy.

NOM with antibiotics represents a promising strategy to reduce resource consumption and avoid unnecessary surgery during the outbreak. The present survey shows that NOM with antibiotics was used routinely in over 50 per cent of patients with uncomplicated appendicitis by only 8·9 per cent of respondents before the pandemic, but currently by 23·7 per cent. Antibiotic management of uncomplicated appendicitis remains uncommon worldwide^28–30^, but RCTs^31–35^ have recently demonstrated that this strategy is safe, with no increased risk of appendiceal perforation and sepsis, and no reported deaths. Although the relapse rate is not negligible, with 27 per cent of patients undergoing appendicectomy within 1 year^36^, these data may be acceptable in the context of an overall strategy during the COVID‐19 outbreak.

Furthermore, a NOM strategy may be implemented as outpatient treatment for uncomplicated appendicitis, with discharge directly from the emergency department after initiation of antibiotic treatment and control of symptoms^34^. In the present survey, the percentage of respondents reporting that their institution treated uncomplicated appendicitis with antibiotics at home and followed up at the outpatient clinic in almost all patients (76–100 per cent) increased from 5·2 per cent before to 9·8 per cent during the pandemic.

A therapeutic strategy based on the shortest possible stay in hospital is highly relevant during the COVID‐19 crisis, as it can reduce the risk of infection and overload of hospitals already stretched by the effects of the outbreak. Safe and effective strategies that allow outpatient antibiotic management of imaging‐confirmed uncomplicated appendicitis are feasible only if established pathways exist to separate patients suspected of having COVID‐19, those who are infected and those who are not^13^. Furthermore, careful evaluation of the clinical presentation and assessment of CT images, and a minimum period of observation in hospital of 6–10 h may be necessary.

A trend towards NOM with antibiotics with or without percutaneous drainage in patients with appendicular abscess was revealed by the survey, with a 7·2 per cent increase in those using it in more than 50 per cent of patients before *versus* during the COVID‐19 pandemic. Conservative treatment of appendicular abscess has been reported to be successful in over 90 per cent of patients, with an overall risk of recurrence of 7·4 per cent and only 19·7 per cent of cases of abscess requiring percutaneous drainage^37^. Conservative treatment has been associated with fewer overall complications (wound infections, postoperative abdominal/pelvic abscesses, ileus/bowel obstructions, and reoperations) than immediate appendicectomy^38^. On the contrary, current evidence shows that surgical treatment is preferable to NOM with antibiotics in reducing duration of hospital stay and need for readmission, especially when laparoscopic expertise is available^39^. A high‐quality randomized trial^40^ demonstrated that laparoscopic appendicectomy in experienced hands is a safe and feasible first‐line treatment for appendiceal abscess; early laparoscopic appendicectomy was associated with fewer readmissions (3 *versus* 27 per cent) and fewer additional interventions (7 *versus* 30 per cent) than conservative treatment, with a comparable duration of hospital stay.

The authors suggest that the laparoscopic approach remains the treatment of choice for complicated appendicitis with abscess, if the patient's clinical condition and the hospital organizational pathways allow safe performance of laparoscopy, with appropriate establishment and management of pneumoperitoneum. Conversely, if management of the COVID‐19 emergency does not allow surgery to be performed safely, NOM could be a reasonable first‐line treatment. Percutaneous drainage as an adjunct to antibiotics, if available, could be beneficial.

This study has limitations. In an effort to collect the largest number of replies, the link was circulated by means of social media, e‐mail lists and via personal contacts. Therefore, the number of recipients cannot be quantified accurately. Using closed questions eased the delivery and rapid analysis of data, and is used in most studies; however, this might have resulted in some information not being captured (such as other hospital settings not reported in question 8). It should be noted that the reported data are estimates based on the best available surgical data from each participating centre. Moreover, respondents in countries where the pandemic was in its earliest stages at the time of survey circulation, such as in Latin America, the UK and the USA, may have underestimated the real impact of COVID‐19 on emergency surgery referrals and operations. The relatively short time since the start of the outbreak could have been insufficient to allow detection of overall changes in decision‐making strategies. However, this study assessed the attitude of surgeons worldwide to a very common disease, and important information can be obtained at a time when sound evidence is lacking. Such data can be useful in identifying adherence to the available guidance statements, and to highlight the priorities that need to be addressed in the near future.

The variation in practice identified by the survey warrants further investigation and should be addressed by international societies globally, ideally by means of joint assessment and preparation of agreed recommendations. The evolving situation calls for guidance to be revised dynamically, as new evidence becomes available.

## Supporting information

Additional supporting information can be found online in the Supporting Information section at the end of the article.

## Supplementary Material

znaa189_Supplementary_Data

## Notes

Members of the ACIE Appy Study Collaborative are co‐authors of this study and are listed in *Appendix S1*, (supporting information)
