# Supplementary material for: Global attitudes in the management of acute appendicitis during COVID‐19 pandemic: ACIE Appy Study
Source: Br J Surg. 2020 Oct 8;108(6):717–26. doi: 10.1002/bjs.11999 (PMC7675377; doi:10.1002/bjs.11999)
Supplement: znaa189_Supplementary_Data [file znaa189_supplementary_data.doc]

**BJS11999**

**Global attitudes in the management of acute appendicitis during COVID-19 pandemic: ACIE Appy Study**

B. Ielpo, M. Podda, G. Pellino, F. Pata, R. Caruso, G. Gravante and S. Di Saverio, on behalf of the ACIE Appy Study Collaborative

**Appendix S1** ACIE Appy Study Collaborative – List of Collaborators

Steering and Writing committee: Benedetto Ielpo, Mauro Podda, Gianluca Pellino, Francesco Pata, Riccardo Caruso, Gianpiero Gravante, Salomone Di Saverio

Dissemination: Gaetano Gallo, Rashid Lui

Participants:

Adam Orengia, Aditya Chowdary, Aditya Kulkarni, Adnan Kuvvetli, Adolfo Navarro, Adolfo Pisanu, Adrian Smith, Adriana Cavero Ibiricu, Aeris Jane D. Nacion, Ahmad Alsaleh, Ahmad Alhazmi, Ahmad Elmabri, Ajaz Wani, Ahmet Rencuzogullari, Aingeru Sarriugarte Lasarte, Ainhoa Valle Rubio, Akshay Bavikatte, Akshay Kumar, Al-radjid Jamiri, Alain Michel Alvarado Padilla, Alban Cacurri, Alberto de San Ildefonso, Alberto Porcu, Alberto Sartori, Aldo Rocca, Alejandro Paz Yáñez, Alejandro Becaria, Alejandro Solís-Peña, Aleksandar Sretenović, Alex Urbistondo, Alfonso Bandin, Alfonso Najar, Alessandro De Luca, Alex Boddy, Alexandros Charalabopoulos, Alexios Tzivanakis, Alfonso Amendola, Alfredo Ramirez-Gutierrez de Velasco, Ali Cihat Yildirim, Alice Frontali, Alpha Oumar Toure, Alvaro García-Granero, Amaia Martínez Roldan, Amaia Sanz Larrainzar, Amila Sanjiva Ratnayake, Ana María Gonzalez-Ganso, Ana M. Minaya-Bravo, Andre Das, Andrea Bondurri, Andrea Costanzi, Andrea Lucchi, Andrea Mazzari, Andrea Musig, Andrea Peloso, Andrea Piano, Andrea Police, Andrei Mihailescu, Andrés Pouy, Angela Romano, Angelo Iossa, Anna Carmen Leonetti, Anna Guariniello, Anna Isaac, Anna Pia Delli Bovi, Antonella Chessa, Antonella Tromba, Antonio Álvarez Martínez, Antonio Brillantino, Antonio Caira, Antonio Castaldi, Antonio Ferronetti, Antonio Giuliani, Antonio Prestera, Antonio Ramos-De la Medina, Antonio Tarasconi, Antonino Tornambè, Arcangelo Picciariello, Argyrios Ioannidis, Ari Leppäniemi, Arshad Khan, Arshad Rashid, Arteaga Luis Eduardo Pérez-Sánchez, Ashok Mittal, Ashrarur Rahman Mitul, Asif Mehraj, Asim Laharwal, Asnel Dorismé, Athanasios Marinis, Atif Iqbal, Augusto Moncada, Bartolomeo Braccio, Basim Alkhafaji, Beatriz de Andrés Asenjo, Beatriz Martin-Perez, Belinda De Simone, Belinda Sánchez Pérez, Ben Creavin, Benedetto Calì, Benedetto Cali, Beniamino Pascotto, Benjamin Stubbs, Benjamin Zavala Retes, Branislav Jovanovic, Brian KP Goh, Bruno Sensi, Carlo Biddau, Carlo Gazia, Carlo Vallicelli, Carlos Alberto Fagundes, Carlos Cerdán Santacruz, Carlos Chirico, Carlos Javier Gómez Díaz, Carlos Petrola, Carlos Sánchez Rodriguez, Carlos Yánez Benítez, Carmelisa Dammaro, Carmelo Lo Faro, Caroline Reinke, Casandra Dominguez Paez, Catalina Oliva, Charudutt Paranjape, Charlotte Thomas, Chi Fung Chia, Chi Kwan Kong, Chiara De Lucia, Christian Ovalle Chao, Claudio Arcudi, Claudio Guerci, Clement Chia, Cristiano Parise, Cristina Folliero, Cristopher Varela, Dalya M. Ferguson, Daniel Camacho, Daniel Popowich, Daniel Souza Lima, Daniela Rega, Daniele Delogu, Daniele Zigiotto, Danilo Vinci, Dario D’Antonio, Dario Parini, David Alessio Merlini, David DE Zimmerman, David Moro-Valdezate, Davide Pertile, Deborah Maria Giusti, Deborah S. Keller, Delko Tarik, Denis Kalivaçi, Dennis Mazingi, Diana Gabriela Maldonado-Pintado, Diego Sasia, Dimitrios Linardoutsos, Dixon Osilli, Domenico Murrone, Domenico Russello, Edgar Rodas, Edisson Alberto Acuña Roa, Edoardo Ricciardi, Edoardo Rosso, Edoardo Saladino, Eduardo Flores-Villalba, Eduardo Ruiz Ajs, Eduardo Smith-Singares, Efstratia Baili, Efstratios Kouroumpas, Eirini Bourmpouteli, Eleftheria Douka, Elena Martin-Perez, Eleonora Guaitoli, Elgun Samadov, Elisa Francone, Elisa Vaterlini, Emilio Morales, Emilio Peña, Enhao Zhao, Eneko Del Pozo Andres, Enrico Benzoni, Enrico Erdas, Enrico Pinotti, Enrique Colás-Ruiz, Erman Aytac, Ernesto Laterza, Ervis Agastra, Esteban Foianini, Esteban Moscoso, Estefania Laviano, Ester Marra, Eugenia Cardamone, Eugenio Licardie, Eustratia Mpaili, Eva Pinna, Evaristo Varo, Fabian Martín Navarro, Fabio Marino, Fabio Medas, Fabio Romano, Fatlum Maraska, Fatmir Saliu, Fausto Madrid, Fausto Rosa, Federica Mastella, Federico Gheza, Federico Luvisetto, Felipe Alconchel, Felipe Monge Vieira, Felipe Pareja, Ferdinando Agresta, Fernanda Luna, Fernando Bonilla, Fernando Cordera, Fernando Burdió, Fernando Mendoza-Moreno, Fernando Muñoz Flores, Fernando Pardo Aranda, Fiona Taylor, Flavia L. Ramos, Flavio Fernandes, Francesca Paola Tropeano, Francesco Balestra, Francesco Bianco, Francesco Ceci, Francesco Colombo, Francesco Di Marzo, Francesco Ferrara, Francesco Lancellotti, Francesco Lazzarin, Francesco Litta, Francesco Martini, Francesco Pizza, Francesco Roscio, Francesco Virdis, Francisco Blanco Antona, Francisco Cervantes Ramírez, Francisco Miguel Fernandez, Francisco Oliver Llinares, Francisco Quezada, Francisco Schlottmann, Fransisco Quezada, Gabriel Herrera-Almario, Gabriel Massaferro, Gabriele Bislenghi, Gabrielle van Ramshorst, Gaetano Gallo, Gaetano Luglio, Georgios Bointas, Georgios Kampouroglou, Georgios Papadopoulos, Gerardo Arredondo Manrique, Giacomo Calini, Giacomo Nastri, Giampaolo Formisano, Giampaolo Galiffa, Gian Marco Palini, Gianluca Colucci, Gianluca Pagano, Gianluca Pellino, Gianluca Vanni, Gianmaria Casoni Pattacini, Gianpiero Gravante, Gilda De Paola, Giorgio Lisi, Giovanna Partida, Giovanni Bellanova, Giovanni De Nobili, Giovanni Sammy Necchi, Giovanni Sinibaldi, Giovanni Tebala, Giulia Bagaglini, Giuliano Izzo, Giulio Argenio, Giuseppe Brisinda, Giuseppe Candilio, Giuseppe Di Grezia, Giuseppe Esposito, Giuseppe Faillace, Giuseppe Frazzetta, Giuseppe La Gumina, Giuseppe Nigri, Giuseppe Romeo, Gloria Chocarro Amatriaín, Gloria Ortega, Gonzalo Martin-Martin, Gregor A. Stavrou, Gunadi, Gustavo Armand Ugon, Gustavo Machain, Gustavo Marcucci, Gustavo Martínez-Mier, Gustavo Miguel Machain, Gustavo Nari, Haydée Calvo, Hamada Fathy, Hamilto, Hazem Ahmed, Hazem Faraj, Hector Nava, Hector Ordas Macias, Herald Nikaj, Heriberto Solano, Huma Ahmed Khan, Humberto Sánchez Alarcón, Husam Ebied, Iacopo Giani, Ibabe Villalabeitia Ateca, Ignacio Neri, Igor Alberdi San Roman, Iliya Fidoshev, Iñaki Martinez Rodriguez, Ionut Negoi, Irene Ortega, Irina Bernescu, Iris Shari Russo, Irune Vincente Rodríguez, Irving Palomares, Isaac Baltazar, Isabel Jaén Torrejimeno, Isabel María Cornejo Jurado, Isabella Reccia, Ishtiyaq Hussain, Ismael Brito Toledo, Ismael Mora-Guzmán, Issam al-Najami, Iulia Dogaru, Ivan Romic, Izaskun Balciscueta, J Cleo Kenington, Jackison Sagolsem, Jae Y Jang, James Olivier, Jan Lammel-Lindemann, Jana Dziakova, Javier Ismael Roldán Villavicencio, Javier Salinas, Jelena Pejanovic Jose Gustavo Parreira, Jovanovic, Jeny Rincón Pérez, Jeryl AS Reyes, Jesus Antonio Medina Luque, Joanna Mak, Joanne Salas Rodriguez, Johnn Henry Herrera Kok, Jon Krook, Jose Antonio Diaz-Elizondo, Jose Castell, José Eduardo García-Flores, José María Jover Navalón, Jose Mauro Silva Rodrigues, José Pereira Pinto, José Tomas Castell Gómez, Juan Bellido Luque, Juan Carlos Martín del Olmo, Juan Carlos Salamea, Juan Francisco Coronel Olivier, Juan Luis Blas Laina, Juliana Maria Ordoñez, Julieta Gutierrez, Julio Abba, Junaid Ahmad Sofi, Kashaf Sherafgan, Kapil Sahnan, Katsuhiko Yanaga, Kevin Beatson, Laharwal Asim, Laura Alvarez, Leandro Siragusa, Lee Farber, Lester Ong, Liarakos Athanasios, Lorena García-Bruña, Luca De Martino, Luca Ferrario, Luca Giordano, Luca Gordini, Luca Pio, Luca Ponchietti, Lucia Moletta, Luciano Curella, Luciano Poggi, Lucio Taglietti, Luigi Bonavina, Luigi Conti, Luigi Goffredi, Luis Angel Garcia Ruiz, Luis Barrionuevo, Luis Enrique Fregoso, Luis F. Cabrera, Luis G Rodriguez, Luis Grande, Luis Gregorio Osoria, Luis Javier Kantun Gonzalez, Luis Sánchez-Guillén, Luis Tallon-Aguilar, Luis Tresierra, Luisa Giavarini, Mahmoud Hasabelnabi, Maja Odovic, Mamoru Uemura, Mansoor Khan, Manuel Artiles-Armas, Mara David, Marcello Di Martino, Marcello Giuseppe Spampinato, Marcelo A. F. Ribeiro Jr, Marcelo Viola, Marco Angrisani, Marco Calussi, Marco Cannistrà, Marco Catarci, Marco Cereda, Marco Conte, Marco Giordano, Marco Pellicciaro, Marco Vito Marino, Maria E Vaterlini, María F. Jiménez, María Giulia Lolli, Maria Irene Bellini, Maria Lemma, Maria Michela Chiarello, Maria Nicola, Mario Arrigo, Mario Caneda Mejia, Mario Montes Manrique, Mario Rodriguez-Lopez, Mario Serradilla-Martín, Mario Zambrano Lara, Marisa Martínez, Mark Bagnall, Mark Peter, Marta Cañón Lara, Marta Jimenez Gomez, Marta Paniagua-Garcia-Señorans, Marta Perez Gonzalez, Martin Rutegård, Martin Salö, Marzia Franceschilli, Massimiliano Silveri, Massimiliano Veroux, Massimo Pezzulo, Matteo Nardi, Matteo Rottoli, Matti Tolonen, Mauricio Pedraza Ciro, Mauricio Zuluagua, Maurizio Cannavò, Maurizio Cervellera, Maurizio Iacobone, Mauro Montuori, Mauro Podda, Melody García Domínguez, Meltem Bingol-Kologlu, Mian Tahir, Michael Lim, Michael Sj Wilson, Michael Wilson, Michela Campanelli, Michele Bisaccia, Michele De Rosa, Michele Maruccia, Michele Paterno, Michele Pisano, Michele Torre, Michelle Treviño, Michele Zuolo, Miguel A. Hernandez Bartolome, Miguel Farina, Miguel Pera, Mikel Prieto Calvo, Milagros Sotelo, Min Myat Thway, Mohamed Hassan, Mohamed Salah Eldin Hassan, Mohammad Azfar, Mohammad Bouhuwaish, Mohammad Taha, Mohammad Zaieem, Mohammed Korkoman, Montserrat Guraieb, Mostafa Shalaby, Muhammad Asif Raza, Muhammad Umar Younis, Muhammed Elhadi, Mujahid Zulfiqar Ali, Nadeem Quazi, Nagendra N. Dudi-Venkata, Nahar Alselaim, Natasha Loria, Nathalie Villan Ramírez, Nay Win Than, Neil Smart, Nelson Trelles, Nicanor Pinto, Niccolò Allievi, Niccolo Petrucciani, Nicola Antonacci, Nicola Cillara, Nicola de'Angelis, Nicolae Gica, Nicolaescu Diana Cristiana, Nicolás Krystek, Nicolò Falco, Nicolò Pecorelli, Nicolò Tamini, Nikolaos Andreas Dallas, Nikolaos Machairas, Noelia Brito, Nura Ahmed Fieturi, Nuria Ortega, Octavio Avila Mercado, Oktay Irkorucu, Omar Alsherif, Orestes Valles, Orestis Ioannidis, Oscar Hernández Palmas, Oscar Isaac Hernandez Palmas, Oscar Sanz Guadarrama, Osman Bozbiyik, Pablo Omelanczuk, Pablo Ottolino, Pablo Rodrigues, Pablo Ruiz, Paola Campenni, Paola Chiarade, Paola Prieto Olivares, Paolo Baroffio, Paolo Panaccio, Pascal Wintringer, Pasquale Di Fronzo, Pasquale Talento, Pasqualino Favoriti, Patricia Sendino, Patrizia Marsanic, Patricia Mifsut, Paúl Andrade, Pawel

**Valentina Ferri**, **Giuseppe** **Massimiliano de Luca, Sara Ingallinella, Eva Pueyo, Francesco Palmieri, Jesus Silva, Ken Min Chin, Nicholas Syn, Brian K. P. Goh, Ye Xin Koh, Valeria Tonini, Ana Gonzales-Ganso, Vicente Simó, Maria Victoria Diago.**

Ajawin, Pedro Abadía-Barnó, Pedro Alfonso Najar Castañeda, Pedro Omar Sillas Arevalos, Pedro Palazón Bellver, Peng Soon Koh, Petry Souza, Piotr Major, Rajandeep Singh Bali, Rakesh Mohan Khattar, Renato Bessa Melo, Reza Ebrahiminia, Ricardo Azar, Ricardo López Murga, Riccardo Caruso, Riccardo Pirolo, Richard Brady, Richard Justin Davies, Rishi Dholakia, Rishi Rattan, Rishi Singhal, Robert Lim, Roberta Angelico, Roberta Maria Isernia, Roberta Tutino, Roberto Faccincani, Roberto Peltrini, Rocio Carrera-Ceron, Rodrigo Tejos, Rohit Kashyap, Roosevelt Fajardo, Rosa Lozito, Royer Madariaga Pareja, Sabrina Garbarino, Salomone Di Saverio, Salvador Morales-Conde, Sami Benli, Sami Mansour, Samir Flores, Samuel Limon Suarez, Santiago Lopez Ben, Sara Fuentes, Sara Gortazar de las Casas, Sara Napetti, Sara Ortiz de Guzmán, Selmy Awad, Sergio A. Weckmann Luján, Sergio Gentilli, Sergio Grimaldi, Sergio Olivares Pizarro, Serkan Tayar, Shakeeb Nabi, Shannon M Chan, Sheikh Junaid, Sidney Rojas, Silvana Monetti, Silvia García, Silvia Salvans, Silvia Tenconi, Simon Shaw, Simone Santoni, Sofia Andrea Parra, Sofía Cárdenas, Sonia Pérez-Bertólez, Sonja Chiappetta, Sophie Dessureault, Spiros Delis, Stefano Amore Bonapasta, Stefano Rausei, Stefano Scaringi, Sundeep Keswani, Syed Muhammad Ali, Süleyman Cetinkunar, Tak Lit Derek Fung, Tariq Rawashdeh ,Tatiana Nicolás López, Tercio De Campos, Teresa Calderon Duque, Teresa Perra, Theodore Liakakos, Theodoros Daskalakis, Theodoros Liakakos, Thomas Barnes, Tijmen Koëter, Tiku Zalla, Tomás E. González, Tomás Elosua, Tommaso Campagnaro, Tommy Brown, Topi Luoto, Touré Alpha Oumar, Ugo Giustizieri, Ugo Grossi, Umberto Bracale, Uriel Rivas, Valentina Sosa, Valentina Testa, Valeria Andriola, Valeria Tonini, Valerio Balassone, Valerio Celentano, Valerio Progno, Varun Raju, Vanessa Carroni, Venera Cavallaro, Venkateswara Rao Katta, Veronica De Simone, Vicent Primo Romaguera, Victor Hugo García Orozco, Victor Luraschi,Victor Rachkov, Victor Turrado-L, Victor Visag-Castillo, Victoria Dowling, Victoria Graham, Vincenzo Papagni, Vincenzo Vigorita, Vinicius Cordeiro Fonseca, Virginia Jimenez Carneros, Vittoria Bellato, Walyson Gonçalves, William F. Powers, William Grigg, Wolf O. Bechstein, Yu Bing Lim, Yuksel Altinel, Zoran Golubović, Zutoia Balciscueta

**Appendix S2** Subgroup analyses

| **Screening patients with appendicitis for SARS-CoV-2** | | | |
| --- | --- | --- | --- |
|  | **Yes, all patients** | **Only patients with respiratory symptoms or suspected of SARS-CoV-2 infection** | **No** |
| **General** | 37.4% | 51.0% | 11.6% |
| **USA** | 17.2% | 65.5% | 17.2% |
| **UK** | 66.7% | - | 33.3% |
| **Spain** | 53.6% | 39.3% | 7.1% |
| **Mexico** | 14.1% | 50.0% | 35.9% |
| **Italy** | 57.1% | 39.0% | 3.8% |
| **France** | 26.3% | 57.9% | 15.8% |
| **Brazil** | 40.0% | 46.7% | 13.3% |
| **India** | 4.5% | 86.4% | 9.1% |

| **Type of screening** | | | | | | | | | |
| --- | --- | --- | --- | --- | --- | --- | --- | --- | --- |
|  | **Chest X-ray** | **Chest X-ray + Serology** | **Chest X-ray + PCR** | **Chest CT scan** | **Chest CT scan + serology** | **Chest CT scan + PCR** | **Serology** | **PCR** | **Rapid test** |
| **General** | 7.3% | 6.3% | 19.8% | 13.9% | 6.7% | 18.1% | 1.4% | 17.2% | 9.3% |
| **USA** | - | 8.3% | 12.5% | - | - | 4.2% | 4.2% | 50.0% | 20.8% |
| **UK** | 4.9% | 2.4% | 9.8% | 36.6% | 12.2% | 26.8% | - | 4.9% | 2.4% |
| **Spain** | 6.5% | 0.9 | 29.6% | 24.1% | 2.8% | 17.6% | - | 16.7% | 1.9% |
| **Mexico** | 29.2% | 6.3% | 10.4% | 16.7% | 4.2% | 16.7% | - | 10.3% | 6.2% |
| **Italy** | 4.4% | 11.7% | 18.0% | 6.8% | 9.2% | 18.4% | 2.4% | 12.1% | 17.0% |
| **France** | - | - | - | 27.8% | 5.6% | 38.9% | 5.6% | 5.6% | 16.5% |
| **Brazil** | - | - | - | 69.2% | - | 23.1% | - | 7.7% | - |
| **India** | 18.2% | 13.6% | 40.9% | - | 4.5% | 9.2% | - | 13.6% | - |

| **Changes in operative protection** | | | | | | |
| --- | --- | --- | --- | --- | --- | --- |
| **Did you change your operative protection in COVID-19 negative patients?** | | | | | | |
|  | **No changes** | **Face mask (FFP2/FFP3)** | **N95 Face mask** | **Goggles** | **Face mask (FFP2/FFP3) and goggles** | **N95 mask and goggles** |
| **General** | 37.9% | 10.2% | 6.4% | 3.4% | 24.0% | 18.0% |
| **USA** | 31.0% | - | 20.7% | - | 6.9% | 41.4% |
| **UK** | 19.0% | 4.8% | 2.4% | 4.8% | 59.5% | 9.5% |
| **Spain** | 31.6% | 12.3% | 1.8% | 7.0% | 39.5% | 7.8% |
| **Mexico** | 19.0% | 6.3% | 14.3% | 1.6% | 9.5% | 49.2% |
| **Italy** | 42.9% | 15.2% | 5.2% | 2.9% | 28.6% | 5.2% |
| **France** | 31.6% | 21.1% | 10.5% | 10.5% | 21.1% | 5.3% |
| **Brazil** | 53.3% | - | - | - | 6.7% | 40% |
| **India** | 13.6% | 18.2% | 9.1% | - | 18.2% | 40.9% |
| **Did you change your operative protection in COVID-19 untested patients?** | | | | | | |
| **General** | 18.1% | 10.6% | 6.0% | 2.4% | 40.1% | 22.6% |
| **USA** | 13.8% | - | 27.6% | - | 6.9% | 51.7% |
| **UK** | 5.0% | 7.5% | 2.5% | - | 72.5% | 12.5% |
| **Spain** | 14.9% | 10.5% | 0.9% | 4.4% | 53.8% | 10.5% |
| **Mexico** | 14.1% | 7.8% | 14,1% | 1.6% | 10.9% | 51.6% |
| **Italy** | 12.0% | 16.3% | 5.7% | 3.3% | 58.9% | 5.7% |
| **France** | 31.6% | 21.1% | 5.3% | 10.5% | 5.3% | 5.3% |
| **Brazil** | 26.7% | - | - | - | 13.3% | 60% |
| **India** | - | 22.7% | 9.1% | - | 22.7% | 45.5% |
| **Did you change your operative protection in COVID-19 positive patients?** | | | | | | |
| **General** | 4.1% | 4.3% | 1.9% | 0.4% | 56.3% | 33.0% |
| **USA** | - | - | 17.2% | - | 10.3% | 72.4% |
| **UK** | 2.4% | 2.4% | - | - | 76.2% | 19.0% |
| **Spain** | - | - | - | 1.8% | 81.6% | 13.2% |
| **Mexico** | 7.8% | 25.0% | - | - | - | 67.2% |
| **Italy** | 3.8% | 9.6% | 0.5% | - | 77.9% | 8.2% |
| **France** | - | 15.8% | - | - | 78.9% | 5.3% |
| **Brazil** | 6.7% | - | 6.7% | - | 6.7% | 80% |
| **India** | - | 4.8% | - | - | 23.8% | 71.4% |

| **Management of uncomplicated (no abscess) acute appendicitis during COVID-19 pandemic** | | | | |
| --- | --- | --- | --- | --- |
|  | **Non-operative management with antibiotics** | **Case-by-case decision** | **Straightforward laparoscopic appendectomy** | **Straightforward open appendectomy** |
| **General** | 23.7% | 38.8% | 22.5% | 15.0% |
| **USA** | 10.3% | 37.9% | 48.3% | 3.4% |
| **UK** | 59.5% | 23.8% | 9.5% | 7.1% |
| **Spain** | 11.4% | 35.1% | 41.2% | 12.3% |
| **Mexico** | 3.1% | 35.9% | 21.9% | 39.1% |
| **Italy** | 39.3% | 50.2% | 8.1% | 2.4% |
| **France** | 15.8% | 47.4% | 36.8% | - |
| **Brazil** | 6.7% | 46.7% | 13.3% | 33.3% |
| **India** | 68.2% | 18.2% | - | 13.6% |

| **Management of complicated (abscess) acute appendicitis during COVID-19 pandemic** | | | | |
| --- | --- | --- | --- | --- |
|  | **Non-operative management with antibiotics** | **Non-operative management with antibiotics + percutaneous drainage** | **Straightforward laparoscopic appendectomy** | **Straightforward open appendectomy** |
| **General** | 5.3% | 32.9% | 33.7% | 28.1% |
| **USA** | - | 82.8% | 13.8% | 3.4% |
| **UK** | 5.0% | 50.0% | 7.5% | 37.5% |
| **Spain** | 6.1% | 47.4% | 37.7% | 8.8% |
| **Mexico** | - | 14.1% | 28.1% | 57.8% |
| **Italy** | 8.1% | 13.4% | 50.2% | 28.2% |
| **France** | - | 15.8% | 73.7% | 10.5% |
| **Brazil** | - | 53.3% | 13.3% | 33.3% |
| **India** | 9.1% | 50.0% | 4.5% | 36.4% |

| **Changes in surgical approach to acute appendicitis during COVID-19 pandemic** | | | |
| --- | --- | --- | --- |
|  | **No** | **Yes, from laparoscopic to open** | **Yes, from open to laparoscopic** |
| **General** | 61.0% | 36.6% | 2.4% |
| **USA** | 82.8% | 13.8% | 3.4% |
| **UK** | 40.5% | 59.5% | - |
| **Spain** | 73.7% | 25.4% | 0.9% |
| **Mexico** | 50.0% | 45.3% | 4.7% |
| **Italy** | 67.1% | 31.9% | 1.0% |
| **France** | 84.2% | 10.5% | 5.3% |
| **Brazil** | 23.1% | 76.9% | - |
| **India** | 40.9% | 54.5% | 4.5% |

| **Confidence in performing open appendectomy** | | | |
| --- | --- | --- | --- |
|  | **No** | **Yes, supervised** | **Yes, unsupervised** |
| **General** | 7.8% | 15.8% | 76.4% |
| **USA** | 3.4% | 6.9% | 89.7% |
| **UK** | - | 9.5% | 90.5% |
| **Spain** | 12.3% | 9.6% | 78.1% |
| **Mexico** | 6.3% | 12.5% | 81.3% |
| **Italy** | 9.0% | 28.4% | 62.6% |
| **France** | 26.3% | - | 73.7% |
| **Brazil** | 13.3% | - | 86.7% |
| **India** | - | 9.1% | 90.9% |

| **Appendectomy on COVID-19 positive patients** | | | | |
| --- | --- | --- | --- | --- |
|  | **Always open (personal preference)** | **Laparoscopic surgery without smoke evacuation systems** | **Laparoscopic surgery with smoke evacuation systems** | **Would use laparoscopy, but smoke evacuation systems unavailable** |
| **General** | 30.1% | 6.2% | 43.0% | 20.7% |
| **USA** | 20.7% | 3.4% | 72.4% | 3.4% |
| **UK** | 45.2% | 11.9% | 31.0% | 11.9% |
| **Spain** | 17.7% | 2.7% | 69.0% | 10.6% |
| **Mexico** | 32.8% | 6.3% | 21.9% | 39.1% |
| **Italy** | 21.5% | 9.8% | 44.4% | 24.4% |
| **France** | 5.6% | 16.7% | 77.8% | - |
| **Brazil** | 60.0% | - | 26.7% | 13.3% |
| **India** | 72.7% | - | 9.1% | 18.2% |

| **Appendectomy on COVID-19 untested patients** | | | | |
| --- | --- | --- | --- | --- |
|  | **Always open (personal preference)** | **Laparoscopic surgery with smoke evacuation systems** | **Would use laparoscopy, but smoke evacuation systems unavailable** | **Would use laparoscopy, but hospital policy does not allow it** |
| **General** | 28.0% | 49.4% | 17.0% | 5.6% |
| **USA** | 10.3% | 79.3% | 10.4% | - |
| **UK** | 34.1% | 39.0% | 7.3% | 19.5% |
| **Spain** | 17.5% | 66.7% | 12.3% | 3.5% |
| **Mexico** | 32.8% | 32.8% | 26.6% | 7.8% |
| **Italy** | 21.3% | 55.1% | 19.8% | 3.9% |
| **France** | 5.3% | 83.7% | - | 11.0% |
| **Brazil** | 40.0% | 46.7% | 13.3% | - |
| **India** | 72.7% | 9.1% | 13.6% | 4.5% |

| **Use of smoke filtrating systems during laparoscopic appendectomy** | | | | |
| --- | --- | --- | --- | --- |
|  | **Yes** | **Only in COVID-19 positive patients** | **Only in COVID-19 positive or untested patients** | **No** |
| **General** | 37.8% | 11.9% | 24.3% | 26.0% |
| **USA** | 58.6% | - | 27.6% | 13.8% |
| **UK** | 66.7% | 4.8% | 7.1% | 21.4% |
| **Spain** | 55.8% | 5.3% | 24.8% | 14.2% |
| **Mexico** | 20.6% | 4.8% | 31.7% | 42.9% |
| **Italy** | 36.5% | 14.9% | 24.5% | 24.0% |
| **France** | 31.6% | 21.1% | 15.8% | 31.6% |
| **Brazil** | 40.0% | 26.7% | 13.3% | 20.0% |
| **India** | 20.0% | 5.0% | 25.0% | 50.0% |

| **Type of smoke filtrating systems** | | | | |  |  |
| --- | --- | --- | --- | --- | --- | --- |
|  | **Commercially available** | **Commercially available with filtration connected to a container with water** | **Commercially available with filtration connected to a sealed container** | **Homemade** | **Homemade with filtration connected to a container with water** | **Homemade with filtration connected to a sealed container** |
| **General** | 32.8% | 7.7% | 22.0% | 11.9% | 14.0% | 11.6% |
| **USA** | 44.4% | 3.7% | 51.9% | - | - | - |
| **UK** | 55.6% | 11.1% | 13.9% | 2.7% | 5.6% | 11.1% |
| **Spain** | 19.5% | 7.5% | 15.0% | 2.8% | 26.2% | 29.0% |
| **Mexico** | 28.1% | 10.5% | 21.1% | 17.5% | 17.5% | 5.3% |
| **Italy** | 40.3% | 7.3% | 20.9% | 13.7% | 9.9% | 7.9% |
| **France** | 47.1% | 5.9% | 17.6% | 17.6% | 5.9% | 5.9% |
| **Brazil** | 14.3% | 7.1% | 28.6% | 21.4% | 21.4% | 7.2% |
| **India** | 28.6% | 21.4% | 21.4% | 14.3% | 7.2% | 7.1% |

**Table S1 Questionnaire as it was circulated**

|  | **1 Baseline Information** |
| --- | --- |
| 1 | Your email address |
| 2 | In which Country are you practicing? |
| 3 | Name of your hospital |
| 4 | Town |
| 5 | What is your level of expertise?   - Student - Consultant/Attending - Fellow - Trainee/Resident |
| 6 | Please, pick the specialty that defines you better   - General and emergency - Colorectal - Upper GI - HPB - Pediatric |
| 7 | In your Country, which kind of National Health System do you have?   - Public health system with universal coverage - Mainly public health system, but without universal coverage - Mainly private health system - Only private health system |
|  | **2. Hospital organisation and screening policies** |
| 8 | Which type of hospital do you work for?   - Public university/teaching hospital - Private university/teaching hospital - Public non-teaching hospital - Private non-teaching hospital |
| 9 | Since COVID-19 pandemic, how did your hospital change its organization?   - My hospital is exclusively dedicated to COVID-19 patients - My hospital has restricted areas dedicated to COVID-19 patients - My hospital does not treat COVID-19 patients |
| 10 | Do you routinely screen patients with appendicitis for SARS-CoV-2 infection before surgery?   - Yes, all patients - Only patients with respiratory symptoms or suspect of SARS-CoV-2 infection - No |
| 11 | If you do, how do you screen patients?   - Chest X-ray - Chest X-ray and serology - Chest X-ray and PCR - Chest CT scan - Chest CT scan and serology - Chest CT scan and PCR - Serology - PCR - Rapid test |
|  | **3. Personal protective equipment PPE** |
| 12 | Did you change your operative protection in COVID-19 negative patients   - No changes - Face mask (FFP2/FFP3) - N95 Face mask - Goggles - Face mask (FFP2/FFP3) and goggles - N95 mask and goggles |
| 13 | Did you change your operative protection in COVID-19 untested patients   - No changes - Face mask (FFP2/FFP3) - N95 Face mask - Goggles - Face mask (FFP2/FFP3) and goggles - N95 mask and goggles |
| 14 | Did you change your operative protection in COVID-19 positive patients   - No changes - Face mask (FFP2/FFP3) - N95 Face mask - Goggles - Face mask (FFP2/FFP3) and goggles - N95 mask and goggles |
|  | **4. Personal attitude: management of acute appendicitis** |
| 15 | Personal attitude: Did you change your attitude in the management of uncomplicated acute appendicitis during the COVID-19 pandemic?   - Yes - Yes, only in COVID+ patients - Yes, only in COVID+ and untested patients - No |
| 16 | Personal attitude: How do you manage uncomplicated (no abscess) acute appendicitis before COVID-19 pandemic?   - Non-operative management with antibiotics - Case-by-case decision - Straightforward laparoscopic appendectomy - Straightforward open appendectomy |
| 17 | Personal attitude: How do you manage uncomplicated (no abscess) acute appendicitis during COVID-19 pandemic?   - Non-operative management with antibiotics - Case-by-case decision - Straightforward laparoscopic appendectomy - Straightforward open appendectomy |
| 18 | Personal attitude: How do you manage complicated (abscess) acute appendicitis before COVID-19 pandemic?   - Non-operative management with antibiotics - Non-operative management with antibiotics and percutaneous drainage - Straightforward laparoscopic appendectomy - Straightforward open appendectomy |
| 19 | Personal attitude: How do you manage complicated (abscess) acute appendicitis during COVID-19 pandemic?   - Non-operative management with antibiotics - Non-operative management with antibiotics and percutaneous drainage - Straightforward laparoscopic appendectomy - Straightforward open appendectomy |
| 20 | Personal attitude: Did you change your attitude in the management of complicated (abscess) acute appendicitis during COVID-19 pandemic?   - Yes - Only in COVID+ patients - No |
| 21 | Personal attitude: Would you change your overall attitude (surgery vs NOM with antibiotics) if you could test all patients?   - I already test all patients - Only if quick test or PCR were available - Yes - No |
| 22 | Personal attitude: During the COVID-19 pandemic, did you change the surgical approach (open vs laparoscopic) to appendectomy?   - No - Yes, from laparoscopic to open - Yes, from open to laparoscopic |
| 23 | Personal attitude: Would you say that you are confident in performing open appendectomy?   - No - Yes, supervised by someone senior or with experience in open appendectomy - Yes, unsupervised |
| 24 | Personal attitude: How are you operating on COVID-19 positive patients with appendicitis?   - Always open surgery, personal preference - Laparoscopic surgery without specific devices for protection and smoke evacuation - Laparoscopic surgery with specific devices for protection and smoke evacuation - I would use laparoscopic, but I do not have devices for pneumoperitoneum/smoke evacuation |
| 25 | Personal attitude: How are you operating on COVID-19 untested patients with appendicitis?   - Always open surgery, I prefer - Laparoscopic surgery - I would use laparoscopic, but I do not have devices for pneumoperitoneum/smoke evacuation - I would use laparoscopic, but hospital policy does not allow it |
| 26 | Personal attitude: If laparoscopic appendectomy is performed, do you use any filter system?   - Yes - Yes, only in COVID-19 positive patients - Yes, only in COVID-19 positive or untested patients - No |
| 27 | Personal attitude: If any evacuation system with filters is used, which type of device do you use?   - Commercially available - Commercially available with filtration connected to a container with water - Commercially available with filtration connected to a sealed container - Homemade - Homemade with filtration connected to a container with water - Homemade with filtration connected to a sealed container |
|  | **5. Patient presentation before and during the pandemic** |
| 28 | Did any patient referred for acute appendicitis test positive for SARS-CoV-2 before surgery at your hospital (percentage)?   - 0% - 1-5% - 6-10% - >10% |
| 29 | Did any COVID-19 negative patient referred for acute appendicitis later test positive for SARS-CoV-2 at your hospital (percentage?)   - 0% - 1-5% - 6-10% - > 10% |
| 30 | How many patients with acute appendicitis are referred to your hospital in one month (before COVID-19 pandemic)?   - < 5 - 5-9 - 10-20 - > 20 |
| 31 | How many patients with acute appendicitis have been referred to your hospital during the last month (during COVID-19 pandemic?)   - < 5 - 5-9 - 10-20 - > 20 |
| 32 | Would you agree that patients admitted with acute appendicitis had more aggressive presentation during COVID-19 pandemic?   - Yes - No - Unsure |
| 33 | In percentage, in how many patients with uncomplicated acute appendicitis (no abscess) a non-operative management with antibiotics is usually used at your hospital (before COVID-19 pandemic)?   - ≤ 25% - 26-50% - 51-75% - 76-100% |
| 34 | In percentage, in how many patients with uncomplicated acute appendicitis (no abscess) a non-operative management with antibiotics is currently used at your hospital (during COVID-19 pandemic)?   - ≤ 25% - 26-50% - 51-75% - 76-100% |
| 35 | In percentage, how many patients with uncomplicated acute appendicitis (no abscess) treated conservatively with antibiotics were sent home and followed-up at the outpatient clinic at your hospital before COVID-19 pandemic?   - ≤ 25% - 26-50% - 51-75% - 76-100% |
| 36 | In percentage, how many patients with uncomplicated acute appendicitis (no abscess) treated conservatively with antibiotics are currently sent home and followed-up at the outpatient clinic at your hospital (during COVID-19 pandemic)?   - ≤ 25% - 26-50% - 51-75% - 76-100% |
| 37 | In percentage, how many patients with complicated acute appendicitis (with abscess) undergo conservative treatment with antibiotics +/- percutaneous drainage at your hospital (before COVID-19 pandemic)?   - ≤ 25% - 26-50% - 51-75 - 76-100% |
| 38 | In percentage, how many patients with complicated acute appendicitis (with abscess) currently undergo conservative treatment with antibiotics +/- percutaneous drainage at your hospital (during COVID-19 pandemic)?   - ≤ 25% - 26-50% - 51-75% - 76-100% |
| 39 | In percentage, how many patients with acute appendicitis treated with surgery undergo open appendectomy at your hospital (before COVID-19 pandemic)?   - ≤ 25% - 26-50% - 51-75% - 76-100% |
| 40 | In percentage, how many patients with acute appendicitis treated with surgery currently undergo open appendectomy at your hospital (during COVID-19 pandemic)?   - ≤ 25% - 26-50% - 51-75% - 76-100% |

**Table S2** Summary of results

| **1. Baseline information** | | | | |
| --- | --- | --- | --- | --- |
| **Query** | **N. of respondents** | **Results** | | |
|  | | | | |
| What is your level of expertise? | 705 | Student | | 0.5% |
|  |  | Consultant/Attending | | 69.9% |
|  |  | Fellow | | 6.9% |
|  |  | Trainee/Resident | | 22.7% |
|  |  |  | |  |
| Pick the specialty that defines you better | 708 | General and emergency | | 57.6% |
|  |  | Colorectal | | 22.9% |
|  |  | Upper GI | | 6.3% |
|  |  | HPB | | 9.9% |
|  |  | Pediatric | | 3.3% |
|  |  |  | |  |
| In your Country, which kind of National Health System do you have? | 707 | Public health system with universal coverage | | 75.0% |
|  |  | Manly public health system, but without universal coverage | | 18.0% |
|  |  | Mainly private health system | | 6.0% |
|  |  | Only private health system | | 1.0% |
|  |  |  | |  |
| **2. Hospital organization and screening policies** | | | | |
|  | | | | |
| Which type of hospital do you work for? | 708 | Public university/teaching hospital | | 63.5% |
|  |  | Private university/teaching hospital | | 13.0% |
|  |  | Public non-teaching hospital | | 16.6% |
|  |  | Private non-teaching hospital | | 6.9% |
|  |  |  | |  |
| Since COVID-19 pandemic, how did your hospital change its organization? | 709 | My hospital is exclusively dedicated to COVID-19 patients | | 8.9% |
|  |  | My hospital has restricted areas dedicated to COVID-19 patients | | 83.1% |
|  |  | My hospital does not treat COVID-19 patients | | 8.0% |
|  |  |  | |  |
| Do you routinely screen patients with appendicitis for SARS-CoV-2 infection before surgery? | 708 | Yes, all patients | | 37.4% |
|  |  | Only patients with respiratory symptoms or suspected of SARS-CoV-2 infection | | 51.0% |
|  |  | No | | 11.6% |
|  |  |  | |  |
| If you do, how do you screen patients? | 654 | Chest X-ray | | 7.3% |
|  |  | Chest X-ray and serology | | 6.3% |
|  |  | Chest X-ray and PCR | | 19.8% |
|  |  | Chest CT scan | | 13.9% |
|  |  | Chest CT scan and serology | | 6.7% |
|  |  | Chest CT scan and PCR | | 18.1% |
|  |  | Serology | | 1.4% |
|  |  | PCR | | 17.2% |
|  |  | Rapid test | | 9.3% |
|  |  |  | |  |
| **3. Personal protective equipment** | | | | |
|  | | | | |
| Did you change your operative protection in COVID-19 negative patients? | 709 | No changes | | 37.9% |
|  |  | Face mask (FFP2/FFP3) | | 10.2% |
|  |  | N95 Face mask | | 6.4% |
|  |  | Goggles | | 3.4% |
|  |  | Face mask (FFP2/FFP3) and goggles | | 24.0% |
|  |  | N95 mask and goggles | | 18.0% |
|  |  |  | |  |
| Did you change your operative protection in COVID-19 untested patients? | 705 | No changes | | 18.1% |
|  |  | Face mask (FFP2/FFP3) | | 10.6% |
|  |  | N95 Face mask | | 6.0% |
|  |  | Goggles | | 2.4% |
|  |  | Face mask (FFP2/FFP3) and goggles | | 40.1% |
|  |  | N95 mask and goggles | | 22.6% |
|  |  |  | |  |
| Did you change your operative protection in COVID-19 positive patients? | 702 | No changes | | 4.1% |
|  |  | Face mask (FFP2/FFP3) | | 4.3% |
|  |  | N95 Face mask | | 1.9% |
|  |  | Goggles | | 0.4% |
|  |  | Face mask (FFP2/FFP3) and goggles | | 56.3% |
|  |  | N95 mask and goggles | | 33.0% |
|  |  |  | |  |
| **4. Personal attitude: management of acute appendicitis** | | | | |
|  | | | | |
| Did you change your attitude in the management of uncomplicated acute appendicitis during the COVID-19 pandemic? | 705 | Yes | | 28.5% |
|  |  | Yes, only in COVID+ patients | | 13.2% |
|  |  | Yes, only in COVID+ and untested patients | | 15.6% |
|  |  | No | | 42.7% |
|  |  |  | |  |
| How do you manage uncomplicated (no abscess) acute appendicitis before COVID-19 pandemic? | 705 | Non-operative management with antibiotics | | 6.6% |
|  |  | Case-by-case decision | | 29.0% |
|  |  | Straightforward laparoscopic appendectomy | | 57.2% |
|  |  | Straightforward open appendectomy | | 7.2% |
|  |  |  | |  |
| How do you manage uncomplicated (no abscess) acute appendicitis during COVID-19 pandemic? | 705 | Non-operative management with antibiotics | | 23.7% |
|  |  | Case-by-case decision | | 38.8% |
|  |  | Straightforward laparoscopic appendectomy | | 22.5% |
|  |  | Straightforward open appendectomy | | 15.0% |
|  |  |  | |  |
| How do you manage complicated (abscess) acute appendicitis before COVID-19 pandemic? | 704 | Non-operative management with antibiotics | | 2.4% |
|  |  | Non-operative management with antibiotics and percutaneous drainage | | 21.1% |
|  |  | Straightforward laparoscopic appendectomy | | 62.5% |
|  |  | Straightforward open appendectomy | | 14.0% |
|  |  |  | |  |
| How do you manage complicated (abscess) acute appendicitis during COVID-19 pandemic? | 701 | Non-operative management with antibiotics | | 5.3% |
|  |  | Non-operative management with antibiotics and percutaneous drainage | | 32.9% |
|  |  | Straightforward laparoscopic appendectomy | | 33.7% |
|  |  | Straightforward open appendectomy | | 28.1% |
|  |  |  | |  |
| Did you change your attitude in the management of complicated (abscess) acute appendicitis during COVID-19 pandemic? | 704 | Yes | | 28.3% |
|  |  | Only in COVID+ patients | | 24.6% |
|  |  | No | | 47.1% |
|  |  |  | |  |
| Would you change your overall attitude (surgery versus non-operative management with antibiotics) if you could test all patients? | 703 | I already test all patients | | 17.5% |
|  |  | Only if quick test for PCR were available | | 26.9% |
|  |  | Yes | | 22.2% |
|  |  | No | | 33.4% |
|  |  |  | |  |
| During the COVID-19 pandemic, did you change the surgical approach (open versus laparoscopic) to appendectomy? | 703 | No | | 61.0% |
|  |  | Yes, from laparoscopic to open | | 36.6% |
|  |  | Yes, from open to laparoscopic | | 2.4% |
|  |  |  | |  |
| Would you say that you are confident in performing open appendectomy? | 704 | No | | 7.8% |
|  |  | Yes, supervised by someone senior, or with experience in open appendectomy | | 15.8% |
|  |  | Yes, unsupervised | | 76.4% |
|  |  |  | |  |
| How are you operating on COVID-19 positive patients with appendicitis? | 695 | Always open surgery, personal preference | | 30.1% |
|  |  | Laparoscopic surgery without specific devices for protection and smoke evacuation | | 6.2% |
|  |  | Laparoscopic surgery with specific devices for protection and smoke evacuation | | 43.0% |
|  |  | I would use laparoscopy, but I do not have devices for pneumoperitoneum/smoke evacuation | | 20.7% |
|  |  |  | |  |
| How are you operating on COVID-19 untested patients with appendicitis? | 700 | Always open surgery, personal preference | | 28.0% |
|  |  | Laparoscopic surgery | | 49.4% |
|  |  | I would use laparoscopy, but I do not have devices for pneumoperitoneum/smoke evacuation | | 17.0% |
|  |  | I would use laparoscopy, but hospital policy does not allow it | | 5.6% |
|  |  |  | |  |
| If laparoscopic appendectomy is performed, do you use any filter system? | 695 | Yes | | 37.8% |
|  |  | Yes, only in COVID-19 positive patients | | 11.9% |
|  |  | Yes, only in COVID-19 positive or untested patients | | 24.3% |
|  |  | No | | 26.0% |
|  |  |  | |  |
| If any smoke evacuation system is used, which type of device do you use | 637 | Commercially available | | 32.8% |
|  |  | Commercially available with filtration connected to a container with water | | 7.7% |
|  |  | Commercially available with filtration connected to a sealed container | | 22.0% |
|  |  | Homemade | | 11.9% |
|  |  | Homemade with filtration connected to a container with water | | 14.0% |
|  |  | Homemade with filtration connected to a sealed container | | 11.6% |
|  |  |  | |  |
| **5. Patient presentation before and during the COVID-19 pandemic** | | | | |
|  | | | | |
| Did any patient referred for acute appendicitis test positive for SARS-CoV-2 before surgery at your hospital (percentage)? | 698 | 0% | 68.1% | |
|  |  | 1-5% | 27.5% | |
|  |  | 5-10% | 3.3% | |
|  |  | >10% | 1.1% | |
|  |  |  |  | |
| Did any patient referred for acute appendicitis later test positive for SARS-CoV-2 at your hospital (percentage)? | 691 | 0% | 71.8% | |
|  |  | 1-5% | 21.3% | |
|  |  | 5-10% | 2.8% | |
|  |  | >10% | 4.1% | |
|  |  |  |  | |
| How many patients with acute appendicitis are referred to your hospital in one month (before COVID-19 pandemic)? | 699 | <5 | 13.3% | |
|  |  | 5-10 | 26.9% | |
|  |  | 10-20 | 27.0% | |
|  |  | >20 | 32.8% | |
|  |  |  |  | |
| How many patients with acute appendicitis have been referred to your hospital during the last month (during COVID-19 pandemic)? | 699 | <5 | 39.3% | |
|  |  | 5-10 | 33.5% | |
|  |  | 10-20 | 16.7% | |
|  |  | >20 | 10.5% | |
|  |  |  |  | |
| Would you agree that patients admitted with acute appendicitis had more aggressive presentation during COVID-19 pandemic? | 701 | Yes | 34.3% | |
|  |  | No | 26.5% | |
|  |  | Unsure | 39.2% | |
|  |  |  |  | |
| In percentage, in how many patients with uncomplicated acute appendicitis (no abscess) a non-operative management with antibiotics is usually used at your hospital (before COVID-19 pandemic)? | 701 | <25% | 79.3% | |
|  |  | 26-50% | 11.8% | |
|  |  | 51-75% | 6.6% | |
|  |  | 76-100% | 2.3% | |
|  |  |  |  | |
| In percentage, in how many patients with uncomplicated acute appendicitis (no abscess) a non-operative management with antibiotics is currently used at your hospital (during COVID-19 pandemic)? | 697 | <25% | 60.1% | |
|  |  | 26-50% | 16.2% | |
|  |  | 51-75% | 11.6% | |
|  |  | 76-100% | 12.1% | |
|  |  |  |  | |
| In percentage, how many patients with uncomplicated acute appendicitis (no abscess) treated conservatively with antibiotics were sent home and followed-up at the outpatient clinic at your hospital before COVID-19 pandemic? | 696 | <25% | 78.2% | |
|  |  | 26-50% | 10.9% | |
|  |  | 51-75% | 5.7% | |
|  |  | 76-100% | 5.2% | |
|  |  |  |  | |
| In percentage, how many patients with uncomplicated acute appendicitis (no abscess) treated conservatively with antibiotics are currently sent home and followed-up at the outpatient clinic at your hospital (during COVID-19 pandemic)? | 692 | <25% | 67.5% | |
|  |  | 26-50% | 12.9% | |
|  |  | 51-75% | 9.8% | |
|  |  | 76-100% | 9.8% | |
|  |  |  |  | |
| In percentage, how many patients with complicated acute appendicitis (with abscess) usually undergo conservative treatment with antibiotics +/- percutaneous drainage at your hospital (before COVID-19 pandemic)? | 695 | <25% | 77.3% | |
|  |  | 26-50% | 10.8% | |
|  |  | 51-75% | 5.6% | |
|  |  | 76-100% | 6.3% | |
|  |  |  |  | |
| In percentage, how many patients with complicated acute appendicitis (with abscess) currently undergo conservative treatment with antibiotics +/- percutaneous drainage at your hospital (during COVID-19 pandemic)? | 690 | <25% | 68.4% | |
|  |  | 26-50% | 12.5% | |
|  |  | 51-75% | 9.3% | |
|  |  | 76-100% | 9.8% | |
|  |  |  |  | |
| In percentage, how many patients with acute appendicitis treated with surgery usually undergo open appendectomy at your hospital (before COVID-19 pandemic)? | 698 | <25% | 73.6% | |
|  |  | 26-50% | 8.7% | |
|  |  | 51-75% | 8.6% | |
|  |  | 76-100% | 9.1% | |
|  |  |  |  | |
| In percentage, how many patients with acute appendicitis treated with surgery currently undergo open appendectomy at your hospital (during COVID-19 pandemic)? | 695 | <25% | 53.8% | |
|  |  | 26-50% | 13.7% | |
|  |  | 51-75% | 9.8% | |
|  |  | 76-100% | 22.7% | |
